# Evaluation of clinical quality improvement interventions: feasibility of an integrated approach

**DOI:** 10.1186/s40814-018-0386-1

**Published:** 2019-01-10

**Authors:** Sandeep Reddy, Kevin Mc Namara, Mary Malakellis, Tim Denton, Cathy McDonald, Jane Opie, Andrew Sanigorski, Vincent Versace

**Affiliations:** 1 Deakin School of Medicine, Waurn Ponds, VIC 3216 Australia; 2 Kardinia Health, Belmont, VIC 3216 Australia; 3 Western Victoria Primary Health Network, Geelong, 3220 Australia

**Keywords:** Cardiovascular disease, Primary care, Quality improvement intervention, Evaluation, Integrated model of evaluation

## Abstract

**Background:**

Cardiovascular diseases (CVD) are the largest cause of death and disability in Australia. Australian national guidelines for the primary prevention of CVD recommend that all adults without CVD and aged 45 years or more are screened for their absolute risk of CVD every 2 years. Despite the compelling evidence to address CVD risk, treatment gaps remain and evidence suggests that much of the shortcomings are attributed to the performance of primary care practices. To address this issue, a quality improvement initiative is being implemented in a large urban multidisciplinary primary care practice in the South West region of Victoria, Australia. The key outcome of this intervention will be to increase the use and acceptability of CVD risk assessment guidelines. To ensure the intervention is tracking toward its objectives, a robust monitoring and evaluation framework was established.

**Method/design:**

A novel framework that assimilates key traditional and theory-driven evaluation practices was developed to assess the impact of the intervention. The framework approach is termed the integrated model of evaluation (IMoE). Researchers and stakeholders convened several times to discuss and develop the evaluation protocol and align it with the quality intervention. The main objective here is to explore the feasibility of an integrated approach to evaluating clinical quality improvement interventions. The sub-objectives are to test the alignment of the IMoE to clinical quality improvement projects and its ability to derive findings to the satisfaction of stakeholders. The design and establishment of the evaluation approach is discussed in further detail in this article.

**Discussion:**

The novel feature of the IMoE is its emphasis on tracking ‘change’ in practices that lead to quality improvement. This emphasis suits the quality improvement theme of this initiative as identification of change elements and explanation behind change is necessary to sustain and promote quality improvement. The other principle behind development of this model, which emphasises practicality in implementation, is to ensure stakeholders gain greatest value from the commissioning of program evaluation. By incorporating practical components and leaving out esoteric concepts, this approach ensures evaluation can be undertaken in realistic timeframes.

**Ethics approval:**

The quality improvement intervention and evaluation framework received approval from the Deakin University Human Research Ethics Committee (Approval Number: 2017-313).

## Background

Cardiovascular diseases (CVD) are the largest cause of death and disability in Australia. *Australia*’*s Health 2016* advises that 13% of Australia’s deaths are due to coronary heart disease and 3% due to cerebrovascular disease [1]. The majority of this CVD burden is preventable [2]. A large international study of heart disease identified that 53% of CVD risk, on a population level, for myocardial infarction can be attributed to diabetes, smoking and hypertension [3]. Australian national guidelines for the primary prevention of CVD recommend that all adults without CVD and aged 45 years or more are screened for their absolute risk of cardiovascular disease every 2 years [2]. Absolute risk is measured using an adapted version of the New Zealand Framingham-based risk equation that is validated for use in Australians aged 45–74 years [2, 4]. National guidelines recommend immediate treatment with lipid lowering and antihypertensive therapy for high-risk patients in addition to lifestyle modification, and potential use of these medications where a patient is at moderate risk but lifestyle modification is delivering inadequate benefit [2]. The merit of this absolute risk approach to management over a traditional single risk factor approach is increasingly evident [5], and the appropriate use of antihypertensive and lipid lowering treatment has been identified as one of the most cost-effective initiatives that can be implemented on a population level [6].

Despite the compelling evidence to address CVD risk, there is significant evidence that treatment gaps remain in the primary prevention of cardiovascular disease [7, 8]. An estimated 970,000 Australians (13% of 45–74-year olds) at high risk of a CVD event within the next 5 years are not receiving combined blood pressure and lipid-lowering treatments as recommended [9]. Vulnerable populations such as Aboriginal and Torres Strait Islanders, lower socio-economic status (SES) and rural communities are often at increased risk [8]. Much of the treatment gap can be traced to the primary care sector [7]. Medicare statistics from 2008 to 2009 suggest that most eligible patients in general practice do not receive cardiovascular health checks. This is because many primary care clinicians do not implement appropriate systems to identify relevant individuals. The use of absolute risk by clinicians is often as an educational tool rather than as a formal assessment tool, and evidence suggests that risk is underestimated using clinical judgement alone [10]. The importance of this issue is highlighted by calls from both the National Health and Medical Research Council and Health Policy Collaboration to address use of guidelines as a priority [11]. The Australian Primary Care Collaboratives (APCC) have demonstrated unequivocally the impact that systematic quality improvement processes can have on improving the performance of general practice against screening indicators [12].

**Fig. 1:**
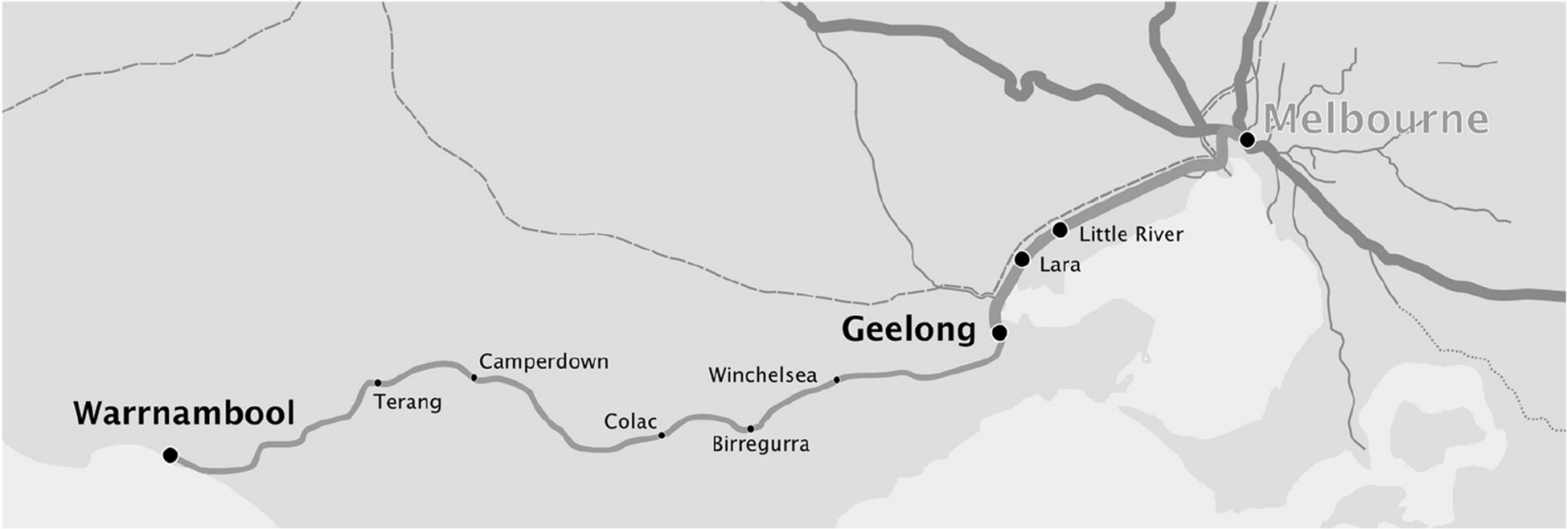
Location of Geelong in South West Victoria, Australia (Image by Marcus Wong, distributed under a CC-BY 2.0 licence)

In this context, a research study to investigate the feasibility of a quality improvement initiative to improve CVD screening has been intitated in a large urban practice (Kardinia Health) in the City of Geelong in the Barwon South West region of Victoria, Australia (see Fig. 1). This research will seek to develop and investigate the feasibility of a continuous quality improvement initiative in primary care, with embedded co-design involving patients and practitioners, on key indicators such as percentage of patients who complete a CVD absolute risk assessment and percent of CVD high-risk patients who are treated appropriately. Additional aims will be to determine the overall impact on cardiovascular health outcomes and equity of impact for key vulnerable groups. Health professionals and patients will be interviewed to inform the process of pre-intervention CVD care to demonstrate current practices, variation in current practices and barriers to achieving use of guidelines in care are understood. Health professionals and patients will be provided with opportunities to reflect on these perspectives, identify key practice challenges and priorities for improvement and select relevant, feasible early goals for improvement. The Plan-Do-Study-Act (PDSA) cycles enables a collaborative approach that will utilise a ‘co-design’ process whereby health professionals and patients are able to contribute to the development and evaluation of quality improvements. To support this process, data extraction is undertaken to capture a broad range of patient information to allow a detailed examination of evidence of CVD treatment gaps across the practice. The quality improvement process is for 24 months with data collection to continue for up to 36 months to determine the sustained impact of the initial quality improvement process on screening performance, after its withdrawal. Protocols will be put in place to ensure open and respectful airing of ideas.

To enable a robust monitoring and evaluation (M&E) framework that tracks the progress and the impact of the quality improvement intervention, the program will adopt an integrated evaluation approach termed ‘integrated model of evaluation (IMoE)’ to guide the M&E process. The integrated approach combines key aspects of traditional and theory-driven evaluation approaches. Traditional evaluation approaches focus mainly on before-after and input-output elements but have been questioned about their validity (internal and external) because of this narrow focus [13–15]. Theory-driven evaluation approaches address this limitation by considering the context in which the intervention occurs and formulating program theory to explain the mechanisms of how interventions work [13, 15]. However, most theory-driven evaluation models take a long time to complete and have other limitations [14, 16]. IMoE seeks to assimilate key elements of traditional ‘method-focused’ and ‘theory-driven’ evaluation practices while presenting a practical evaluation approach that can be implemented in realistic time frames. Also, the emphasis on change in this model closely aligns with the quality improvement intervention. The evaluation process closely aligns with the quality improvement intervention both in terms of time frames and implementation phases. For example, prior to the commencement of the quality improvement intervention, a formative evaluation will be conducted. During the intervention, a process evaluation and following some months after the conclusion of the intervention, a summative evaluation will be conducted.

The main objective of this study is to explore the feasibility of implementing an integrated approach to evaluating clinical quality improvement interventions. The sub-objectives of the study are to:

- ▪ Test the alignment of an integrated evaluation approach, which emphasies quality improvement monitoring and program theory testing, to clinical quality intervention projects
- ▪ Examine if the IMoE approach is adequate to derive findings to assess the outcomes of the intervenion and inform ongoing quality improvement planning in the practice
- ▪ Explore the acceptability of the IMoE approach amongst the primary healthcare workforce as an appropriate tool to monitor and evaluate clinical quality improvement projects.

## Design

### Setting

Kardinia Health is a large primary health care practice, located in the City of Greater Geelong (Barwon South West region of Victoria) that incorporates the values of traditional general practice with multidisciplinary, team-based care and an academic research unit in general practice [17, 18]. Kardinia Health maintains a secured patient database that strictly follows ‘Australian Standards of Best Practice’. As of October 1st, 2018, this restricted access database consisted of 16,904 total patient records with 9963 patients considered active, that is, patients that have attended the practice three times within a 2-year period. There are relatively small proportions of Aboriginal and Torres Strait Islanders (1.25%) and culturally and linguistically diverse (CALD) populations. The combination of academic research with multidisciplinary care at Kardinia Health provides a unique opportunity for the introduction of a quality intervention to improve the prevention of CVD in general practice. To ensure the embedding of processes for improved CVD care, a robust M&E framework was considered important. Following the development of the IMoE model, it was reviewed by the organisation’s research committee and considered appropriate to monitor the quality intervention.

### Design

The key components of the IMoE are as follows:

- Program theory: is a causal statement outlining the expected outcomes as a result of the program intervention in a particular context [13, 15]. They can also be described as a set of assumptions that allows one to understand how an intervention works. Inclusion of program theory in its approach is what mainly separates theory-driven evaluation approaches to traditional evaluation practices [14, 19]. The program theory is developed ahead of the assessment through a consultation with stakeholders, the review of literature and other sources [14]. For the purpose of this model, the stakeholders are the commissioners of the evaluation, the program/project staff and the beneficiaries of the program. Governance structures for some health projects means representatives of such groups could be considered as stakeholders. The program theory is then tested throughout the course of the evaluation using appropriate research methods [13]. Inclusion of the program theory component in the IMoE ensures views of stakeholders are taken into account and a theory is developed about how the program is working or not. The program theory also considers the context in which the program was introduced thus tailoring assessment and solutions to be context specific. By refining or revising the theory later, the evaluation ensures assumptions are tested and appropriate solutions are presented if the program is not working.
- Context: describes the situation in which the program has been introduced and is operating. The context includes geo-political, economic and other scenarios that influence the program’s implementation and outcomes. The context component is often ignored in many generic evaluation approaches exposing the results to poor validity [20]. Programs do not succeed or fail merely because of the resources or change brought in by the program but also because of the context in which they were introduced [13, 14, 21]; ignoring context in assessment lessens the credibility of evaluation results. Therefore, the IMoE includes and emphasises description and assessment of context.
- Intervention: includes the resources and outputs being introduced through the program. Inputs and outputs are data collected through regular evaluations but the IMoE groups them under the ‘intervention category to distinguish it from the “Change” and “Outcomes”’ components in the model. This is necessary not only to highlight the intervention but also present a plausible sequence of events leading to the outcomes as depicted in the program logic and program theory.
- Change: incorporates variations that have occurred as a result of the program intervention. Change is an important outcome in health interventions especially so in quality-focused interventions [21–23]. However, this facet often gets ignored by many of the evaluation approaches. In the IMoE, change (both behavioural and process) is considered as an important process to be tracked. As per the model, suitable indicators are developed to track the changes. The changes can be positive or adverse. Positive changes are those that support program objectives and the adversarial changes are those that deter achievement of program objectives. The changes are to be stated in the preliminary program theory and assessed during the course of the evaluation.
- Outcomes: are the end results of a program, i.e. the objectives or the goals the program set to achieve [15, 20, 24]. Depending on the duration of the program, short-term or mid-term or long-term outcomes are considered in the evaluation. The outcomes are assessed through key-performance indicators (KPIs) developed by the evaluators in consultation with stakeholders. The KPIs can be included in the program theory but this is optional.

**Fig. 2:**
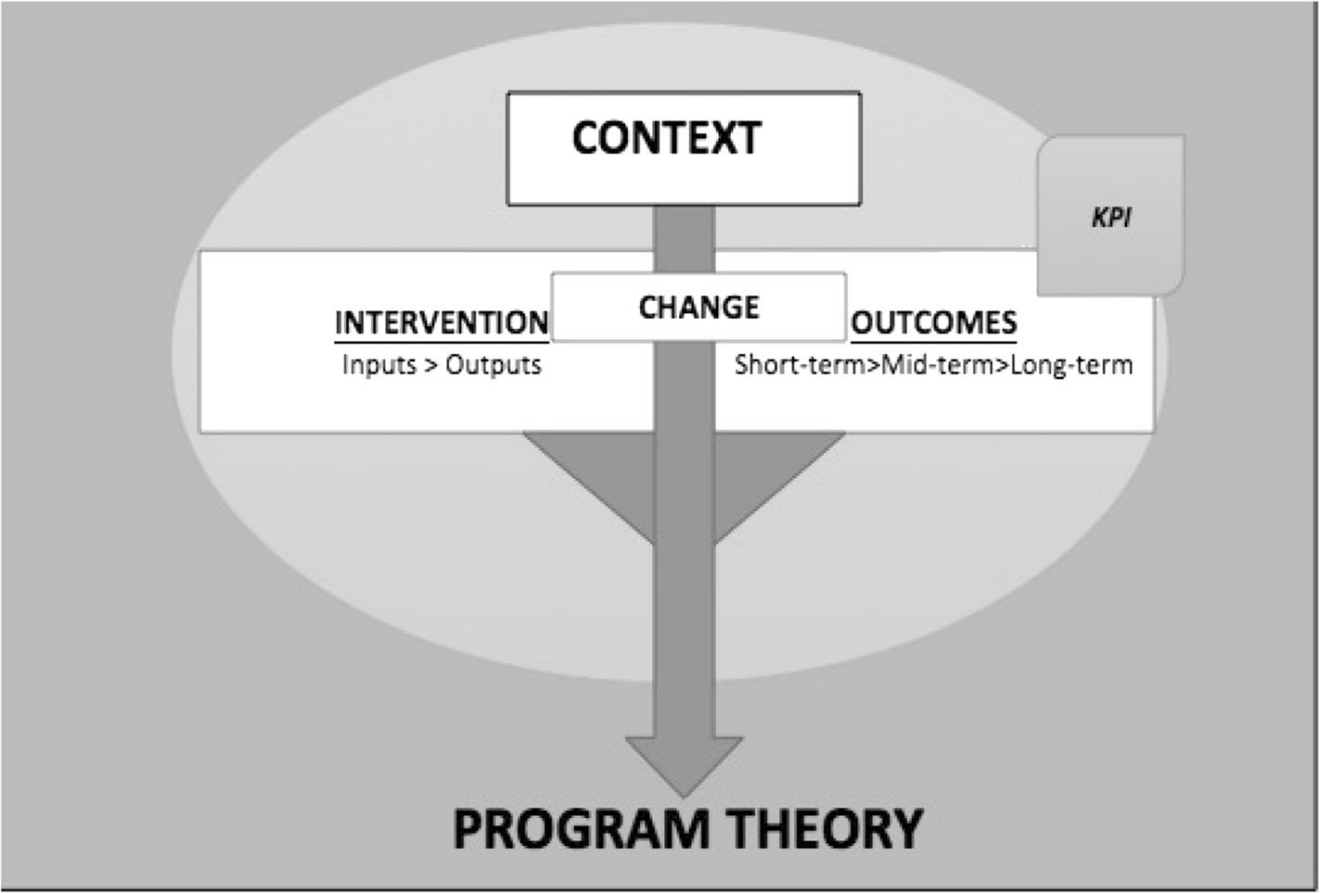
Integrated model of evaluation

The key elements in the IMoE approach are visually represented in Fig. 2 and its contrast to traditional and theory-driven approaches is outlined in Table 1.

**Table 1:** IMoE approach contrasted with traditional and theory-driven evaluation approaches

Traditional evaluation approaches		Theory-driven evaluations		IMoE approach
Advantages	Limitations	Advantages	Limitations	
Generally quicker to complete	Inability to capture contextual factors	Comprehensive assessment of program and context	Takes longer time to complete	Incorporation of essential steps from both approaches to not only ensure a well-rounded but an evaluation completed on time
				Model is relevant to health context because of its emphasis on ‘change’
Process and results easier to understand	Results are incomplete	Results consider the role of contextual factors and outline a causal theory	Process and results are hard for stakeholders to understand	A streamlined and understandable process that involves stakeholders at various levels

### Implementation process

To enable a governance process, Kardinia Health has a steering committee that will oversee the implementation of the IMoE model. The committee’s main role is to advise the evaluators about implementation and receive regular reports about the progress of the evaluation. The evaluation process will complement the quality intervention for the duration of the program. In parallel to the quality intervention, the evaluation will be implemented in three stages: formative, process and summative. The key emphasis of the IMoE model is tracking ‘change’ in practices that lead to quality improvement and this will be reflected in all three stages of the evaluation.

The IMoE approach will commence with a formative evaluation to ensure the quality improvement goals and the resources required to meet these goals are in line with stakeholder’s expectations. The formative evaluation will take into account pre-intervention or baseline key performance indicators (KPIs) to track program progress and impact. In addition to this, a preliminary program theory to explain the change process will be developed. Over the course of the program and as part of the process evaluation, KPI data will be collected to track progress toward program goals. At the end of the program, a summative evaluation to assess the efficacy and efficiency of the program will be undertaken. Also, the preliminary program theory will be revisited to suitably explain the change that has resulted from this intervention. This comprehensive approach will ensure a thorough assessment of the intervention (pre and post) is undertaken.

The availability of high quality, reliable and pertinent data underpins the ability to undertake quality improvement. The main KPIs for the intervention have been identified as follows:

- The percent of active patients aged 45–74 years with all necessary data for CVD absolute risk assessment collected over the past 2 years (with or without CVD)—age, gender, blood pressure, total and HDL cholesterol, diabetes, status smoking status.
- Percent of patients at high risk of CVD or with CVD who are treated appropriately with medications (lipid lowering plus antihypertensive medicines).
- Number of vulnerable patients with CVD risk being screened

In addition to monitoring the collection of absolute cardiovascular risk factor data (diabetes, smoking, blood pressure and cholesterol data), the evaluation will be assessing if modifiable/behavioural risk factors (physical activity, obesity and fruit and vegetable intake) are being screened by clinicians. Literature has indicated complementing screening of cardiovascular absolute risk factors with modifiable risk factors followed by relevant intervention maximises the preventative potential of the intervention [25]. Involvement of clinicians and relevant staff members to adopt appropriate screening practices and counsel relevant patients to engage in healthy behaviour through the quality improvement intervention is the ‘change’ being assessed in this evaluation. The emphasis on monitoring change in this evaluation model means involvement of clinicians and patients (stakeholders and beneficiaries) are key to the implementation of this model.

Indicator data will be extracted from the practice’s patient information system. The practice will develop a register of patients requiring risk screening in accordance with national guidelines. Risk factor monitoring will also be performed with all patients that have been diagnosed with CVD. Data extraction, capturing a broad range of patient information, will also be used to conduct detailed examination of evidence treatment gaps across the practice relating to CVD risk assessment and management.

**Fig. 3:**
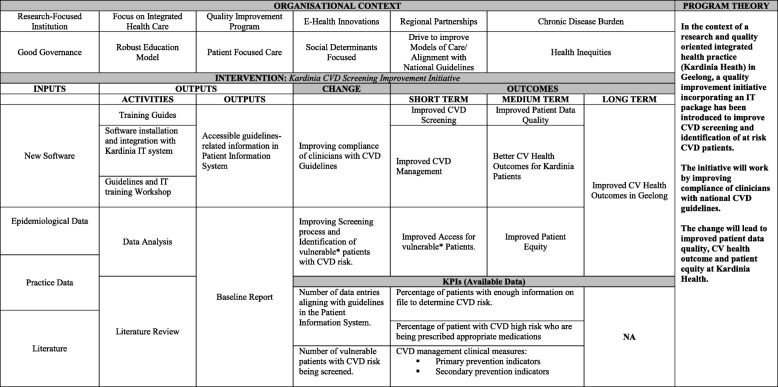
Integrated Program Logic

The integrated program logic for this evaluation, based on the IMoE model, is outlined in Figure 3.

### Program theory

One of the important features of the IMoE approach is the development of and testing of a program theory (causal statement outlining how the program outcomes will be achieved). The preliminary program theory for the quality intervention is outlined here:

In the context of a research and quality oriented integrated health practice **(**Context), a quality improvement initiative involving a collaborative model has been introduced (Intervention) to improve CVD screening and identification of at risk CVD patients. The initiative will work by improving compliance of clinicians with national CVD guidelines (Change). The change will lead to improved patient data quality and CV health outcomes at Kardinia Health (Outcome).

To assess the program theory, semi-structured interviews of Kardinia Health staff and patients coupled with document analysis will be undertaken. A purposive sampling strategy will be used to identify interview patients who are 45–75 years. All staff involved in the strategy group (steering committee), co-design group and project management group (there are three distinct groups) will be invited to participate in the semi-structured interviews. Interviews will be conducted in the formative and summative evaluation stages to assess the validity of the program theory and revise as necessary.

### Analysis

To assess the efficacy of the quality improvement intervention, data from over 9000 patient records will be extracted for analysis by the new software. The KPIs mentioned earlier will be derived from this phase of analysis. For the semi-structured interviews, it is expected that a total number of 22 participants (10 staff and 12 patients) will be available. The 12 patients will be derived from the co-design patient group that has already been established for the clinical quality improvement intervention and the 10 staff will comprise a mix of doctors, nurses, allied health and managers working in the practice. From a qualitative research perspective, this number of participants seems adequate to help with the testing of the program theory [26]. The data from the semi-structured interviews will be thematically analysed to capture how staff’s perspectives have shifted after the intervention. The analysis process will involve transcribing of interviews, initial coding into preliminary codes and aggregation to themes. Following which the themes will be cross-matched with the changes outlined in the IMoE program logic model (Figure 3) and the program theory. The narrative will also be used to test and if necessary revise the preliminary program theory. In addition to interview data, minutes from research and stakeholder meetings and practice reports will be analysed. Based on emerging data, the program theory will either be refined and revised until after the summative evaluation when a final program theory will be confirmed.

To analyse the KPIs pre and post-intervention, univariate comparisons between groups will be conducted using χ^2^ test for equal proportion (or Fisher exact tests where numbers were small) and will be reported as either percentages (*n*) or percentages (95% CI). Continuous normally distributed variables will be compared using Student *t* tests and reported as means (95% CI). Nonparametric data will be compared using Wilcoxon rank-sum tests and reported as medians (interquartile range). Pairwise differences between pre- and post-intervention values will use paired *t* tests for normally distributed data, Hodges-Lehmann median difference estimate for non-normally distributed continuous variables and related-samples McNemar’s test for changes to proportions. Statistical process control techniques will be investigated for appropriateness in confirming real improvements to performance. In essence, this attempts to distinguish any change in performance as a result of quality improvement measures, from underlying trends in changing performance.

### Ethics approval and informed consent

The quality improvement intervention and evaluation framework received approval from the Deakin University Human Research Ethics Committee (Approval Number: 2017-313).

## Discussion

Program evaluation is a well-established methodology to assess the effectiveness and efficiency of programs [15, 20, 24, 27, 28]. The methodology to undertake program evaluations has become diverse and complex over years. In the traditional evaluation approach, the emphasis is on the research methods [20, 24] while in theory-driven approaches, formulation and testing of the program theory is a key process [13, 14, 29]. Because of the emphasis on methods in the traditional approach, the mechanism of the intervention and the reasons why an intervention works (or does not work) is not well understood in this approach [20]. Simplistic representation of input-outputs explained little about the mechanisms of changes and ignored the context in which the change occurred [15, 24]. To address these limitations, theory-driven evaluation approaches that emphasised the development and testing of program theories were introduced [13, 15]. While theory-driven approaches address these forms of limitations, they tend to be difficult to implement, take a long time to complete, require enormous amounts of data and stakeholders may not readily understand the results derived [14, 16]. This is unhelpful as all commissioned evaluations need not only to assess program but also present solutions to issues identified. By presenting abstract results that are unable to be followed up, current theory-driven models may not necessarily benefit stakeholders.

To address inherent limitations of both traditional and theory-driven approaches, the IMoE utilises essential components from both evaluation approaches. The model includes traditional aspects of program evaluation such as program logic components: inputs, outputs, outcomes and KPIs. In addition, it includes theory-driven elements such as contextualisation of the research and formulation of program theory, which are often ignored in traditional program evaluation models at the detriment of the validity of the results. The model also includes innovative elements such as the emphasis on change. This approach we consider is particularly relevant to evaluating this quality intervention. It is known through literature that primary care clinicians find it difficult to incorporate CVD guidelines and risk assessment tools in their practice [7, 9, 10]. This in turn has resulted in poor screening of CVD patients [7]. The intervention being evaluated intends to change this practice such that acceptability of CVD risk screening adheres to national guidelines and is embedded in regular clinical care. As there is considerable emphasis in the IMoE model on tracking this change and testing the efficacy of the intervention, the integrated approach is well placed to inform the M&E framework for this intervention.

The main principle behind development of the integrated model is to ensure stakeholders gain greatest value from the commissioning of program evaluation. By incorporating practical and useful components and leaving out esoteric concepts, the integrated model ensures deployment of this model can be done in realistic time frames. The emphasis of the IMoE is not the methods or academic interests of the evaluator but usefulness of the evaluation results to stakeholders, i.e. are the results valid and can they be acted upon? This is achieved through incorporation of the program theory and emphasis on the context, intervention and change. Also, as there is emphasis on program theory formulation, stakeholders are involved from the very onset. This provides an opportunity for their assumptions to be tested through a rigorous approach. As the IMoE incorporates context in the construction of the program theory, it ensures the results are tailored to the particular program, organisation and scenario. Further, by incorporating program logic elements, the IMoE ensures the implementation is practical, results are understandable to stakeholders and the model is not restricted to impact assessments only.

The main limitation with this study is the first time an integrated approach is being considered to monitor and evaluate clinical quality improvement interventions. It is not known if the model is suitable to such interventions. Hence, one the objectives of this study is to test the alignment. The other caution is about the generasibility of the study findings to other clinical quality improvement interventions. While there is every intention on part of the authors to promote the wide use of the IMoE approach per say, the findings of this evaluation should be reviewed in the context of the Kardinia Health practice environment. Hence, the contextual description in the program theory and program logic to emphasise the prevalent conditions in Kardinia Health. In spite of these limitations, the authors consider the IMoE approach to have several strengths. The IMoE brings together the best of the traditional and theory-driven approaches of program evaluation. While it incorporates several components from both the approaches, the enjoining does not result in a complex or unwieldy model. In fact, a streamlined stakeholder centric process is constructed. To enable wider use of the model following completion of the study at Kardinia Health, the researchers involved in this study will be pursuing the implementation of this approach at other healthcare sites.

## Abbreviations

CVD: Cardiovascular disease
IMoE: Integrated model of evaluation
